# The Effect of Low Dialysate Sodium Concentration on Ambulatory Aortic Blood Pressure and Arterial Stiffness in Patients With Intradialytic Hypertension: A Randomized Crossover Study

**DOI:** 10.7759/cureus.77079

**Published:** 2025-01-07

**Authors:** Fotini Iatridi, Marieta P Theodorakopoulou, Robert Ekart, Artemios G Karagiannidis, Konstantinos Malandris, Efstathios Xagas, Ioanna Revela, Ioannis Tsouchnikas, Panagiotis Giamalis, Pantelis Sarafidis

**Affiliations:** 1 First Department of Nephrology, Hippokration Hospital, Aristotle University of Thessaloniki, Thessaloniki, GRC; 2 Department of Dialysis, Clinic for Internal Medicine, University Medical Center Maribor, Maribor, SVN; 3 Clinical Research and Evidence-Based Medicine Unit, Aristotle University of Thessaloniki, Thessaloniki, GRC; 4 Nephrology, Frontis Dialysis Center, Athens, GRC

**Keywords:** ambulatory blood pressure monitoring, aortic blood pressure, arterial stiffness, dialysate sodium concentration, intradialytic hypertension

## Abstract

Introduction: Intradialytic hypertension (IDH) is associated with increased cardiovascular risk. Arterial stiffness is a strong predictor of adverse outcomes in dialysis patients and may contribute to the development of the phenomenon, as patients with IDH exhibit higher ambulatory aortic blood pressure (BP) and arterial stiffness parameters than patients without IDH.

Methods: This analysis examined the effect of low (137mEq/L) compared to standard (140mEq/L) dialysate sodium concentration on 48-hour aortic BP and arterial stiffness parameters in IDH patients. In this prespecified secondary analysis of a randomized, single-blind, crossover study, 29 IDH patients underwent four hemodialysis sessions with low followed by four sessions with standard dialysate sodium or vice-versa. Mean 48-hour, pre-/post-dialysis and intradialytic aortic systolic/diastolic BP (SBP/DBP), and arterial stiffness indices were assessed.

Results: Mean 48-hour aortic SBP/DBP were significantly lower with low versus standard dialysate sodium (124.1±16.4/83.0±14.1mmHg vs 128.5±12.9/85.8±14.1mmHg, p=0.013/p=0.006 respectively). Low dialysate sodium also significantly reduced pre-dialysis aortic SBP (126.4±17.4 vs 135.6±18.6mmHg, p=0.044) and post-dialysis aortic SBP (137.0±20.0 vs 147.9±18.1mmHg, p=0.01). All wave reflection indices were numerically lower with low dialysate sodium; among them, heart rate-adjusted augmentation index (AIx(75)) was significantly lower during the 48-hour (26.3±6.7 vs 27.7±5.8%, p=0.03), 44-hour, day-time and intradialytic periods. Low dialysate sodium resulted in decreased 48-hour pulse wave velocity (PWV) (9.9±2.5 vs 10.1±2.6m/s, p=0.008); similar differences for PWV were observed during all examined time intervals.

Conclusions: In conclusion, ambulatory 48-hour aortic BP and arterial stiffness parameters were significantly lower using low compared to standard dialysate sodium in IDH patients. These findings further support the use of low dialysate sodium for BP management in this population.

## Introduction

Patients with end-stage kidney disease (ESKD) undergoing hemodialysis have an increased risk for cardiovascular disease, which is the leading cause of mortality accounting for more than 50% of the deaths [1]. Hypertension has an estimated prevalence of approximately 85% and constitutes the most prevalent modifiable cardiovascular risk factor in these individuals [2]. Increased arterial stiffness is a major underlying mechanism of increased blood pressure (BP) and pulse pressure (PP) levels, as well as diastolic cardiac dysfunction in these patients [3]. Previous seminal studies in ESKD patients showed that pulse wave velocity (PWV), a direct measure of arterial stiffness, as well as wave reflection indices such as augmentation pressure (AP) and augmentation index (AIx) are strong and independent predictors of cardiovascular outcomes [4,5]. Additionally, aortic BP displays strong associations with cardiovascular events and mortality and may serve as a superior marker of cardiovascular risk compared to peripheral BP [6,7]. In a prospective cohort study, we showed that ambulatory PWV, AIx and aortic PP were significantly associated with increased risk for cardiovascular events and mortality, whereas ambulatory peripheral BP was not [7]. Finally, previous observations suggest that arterial stiffness parameters in ESKD do not always improve, after a decrease in BP levels, suggesting a BP-dependent and a BP-independent component of this arterial injury [8,9].

The most common pattern of BP trajectory during the intra- and interdialytic interval in the general hemodialysis population is a rapid decrease in BP during hemodialysis, as a response to ultrafiltration, followed by a gradual increase during the interdialytic interval [2,10]. However, approximately 10-15% of the dialysis population, exhibit a “paradoxical” rise in BP during or immediately after dialysis, known as “intradialytic hypertension” (IDH) [11]. Several studies suggest that patients with IDH experience an increased risk for adverse outcomes, compared to those displaying BP fall during hemodialysis [12,13]. Among pathogenetic mechanisms entailed in the development of this phenomenon, volume and sodium overload, overactivation of the renin-angiotensin-aldosterone system (RAAS) and sympathetic nervous system (SNS), as well as endothelial dysfunction and arterial stiffness play prominent roles [11].

A few studies have compared levels of aortic BP and arterial stiffness parameters in patients with and without IDH, showing significantly higher peridialytic [14] and ambulatory [15] wave reflection indices and PWV in IDH patients. A previous study that examined the effect of nebivolol and irbesartan on ambulatory aortic BP and PWV in this population demonstrated significant decreases in these parameters with weekly administration of both drugs [16]. In a recent randomized crossover study, we showed that low dialysate sodium concentration is associated with significantly lower intradialytic, post-dialysis and 48-hour BP levels in IDH patients [17]. To date, no study has evaluated the effects of any non-pharmacological intervention on aortic BP and arterial stiffness in these individuals. Therefore, this analysis aimed to investigate the effect of low (137 mEq/L) compared to standard (140 mEq/L) dialysate sodium concentration on 48-hour ambulatory aortic BP and arterial stiffness parameters in patients with IDH.

## Materials and methods

Study participants

This is a prespecified secondary analysis of a single-blind, randomized, crossover study that examined as primary outcome the effect of low versus standard dialysate sodium concentration on 48-hour ambulatory BP in patients with IDH, as described elsewhere (trial is registered in ClinicalTrials.gov NCT05430438) [17]. The objective of the present analysis was to assess the effect of low versus standard dialysate sodium concentration on 48-hour ambulatory aortic BP and arterial stiffness parameters in patients with IDH. Patients were enrolled from four collaborating Hemodialysis Units, three located in Greece and one in Slovenia, from June 2022 through June 2023. The study protocol was approved by the Ethics Committee of the School of Medicine, Aristotle University of Thessaloniki (Approval Number 4555) and all procedures were performed according to the Declaration of Helsinki (2013 Amendment). Eligibility criteria were: i) adult patients with ESKD on a standard thrice-weekly hemodialysis schedule for more than three months; ii) IDH, defined as SBP rise ≥10 mmHg pre- to post-dialysis for at least four out of six consecutive hemodialysis sessions; iii) patients at dry weight, as assessed by clinical criteria; iv) informed written consent [17]. Exclusion criteria were the following: i) post-dialysis SBP <130 mmHg in at least four out of six consecutive hemodialysis treatments during the selection period prior to study enrollment; ii) presence of old, non-functional, arteriovenous fistula in the arm used for ambulatory blood pressure monitoring (ABPM) that could interfere with proper recording; iii) contraindications for the intervention of low dialysate sodium (e.g., frequent hypotensive episodes requiring fluid resuscitation); iv) pre-dialysis serum sodium <130 mEq/L or >142 mEq/L at recruitment; v) modification of dry weight or antihypertensive medication during one month prior to enrollment; vi) hospitalization for any cause during one month before study enrollment; vii) history of seizures or dialysis disequilibrium syndrome; viii) active malignancy or any comorbidities with poor prognosis [17].

Study procedures

Records of peridialytic BP measurements during a two-week period before study enrollment were assessed to confirm the presence of IDH. Demographic and anthropometric characteristics, medical history, medication, and other dialysis-related parameters were recorded for every participant. During baseline evaluation, performed before a mid-week dialysis session, peridialytic and intradialytic BP were assessed with the Mobil-O-Graph device (IEM, Stolberg, Germany) [17]. Intradialytic BP corresponds to the mean of all recordings obtained every 20 minutes during dialysis, without including pre/post-dialysis recordings. Subsequently, patients were randomly assigned to two groups, which received the two interventions in the opposite order [17]. Block randomization with a block size of four, stratified by gender, was used to determine treatment order based on a computer-generated randomization list. An independent member of our research group, who was not involved in the rest of study procedures and assessments, revealed the randomization arm for each participant to the respective investigator of each dialysis unit, after patients completed their baseline evaluation, via written communication. Group A received low, i.e. 137 mEq/L, dialysate sodium followed by standard, i.e. 140 mEq/L dialysate sodium concentration, while Group B received standard followed by low dialysate sodium concentration. All participants underwent four hemodialysis sessions, starting from a mid-week session (i.e. Wednesday or Thursday), with low or standard dialysate sodium concentration, according to the randomization arm. After a two-week washout period with standard dialysate sodium of 140 mEq/L for both groups, participants underwent another set of four hemodialysis sessions with the opposite dialysate sodium concentration [17]. 

Ultrafiltration volume was determined based on the participants’ prespecified dry weight, defined according to standard clinical criteria. Modification of dry weight or antihypertensive medication was not allowed during the study. Participants were advised to follow their customary daily routines, encompassing physical activity, food and water intake, as well as to adhere to their prescribed medications throughout the duration of the study [17].

Assessments

Ambulatory brachial and aortic BP and PP, indices of wave reflection [AIx, defined as the ratio of AP to aortic PP and heart rate-adjusted AIx (AIx(75)], and PWV were evaluated with the Mobil-O-graph NG monitor, a validated oscillometric ABPM device [18]. After recording brachial BP, the cuff is re-inflated for approximately 10 seconds and records the brachial pulse waves using a high-fidelity pressure sensor (MPX5050, Freescale, Temple, AZ, USA). Brachial systolic BP (SBP) and diastolic BP (DBP) are used for calibration of the brachial pulse waveforms, while the aortic pulse waveform is generated by the software (HMS version 5.1) using an ARCSolver algorithm with generalized transfer function. Wave separation analysis is performed by decomposing the aortic pulse waveform into forward (incident) and backward (reflected) pulse waves with a triangular aortic flow waveform [10]. The generated aortic pulse wave is used for the pulse wave analysis, while PWV is estimated via mathematical models assuming a three-element Windkessel model and taking into account the aortic characteristic impedance, aortic SBP and age [19,20]. Previous validation studies in hemodialysis patients showed acceptable agreement between Mobil-O-Graph-derived parameters and measurements obtained by Sphygmocor (AtCor, Sydney, Australia).

The device was placed on the non-fistula arm with cuffs of appropriate size and was set to measure BP every 20 minutes during day-time (7:00 to 22:59) and every 30 minutes during night-time (23:00 to 6:59). The recording began immediately before the start of the fourth session (mid-week) on each dialysate sodium and lasted for a complete 48-hour period, that included the four-hour intradialytic period and the 44-hour interdialytic period [17]. Peridialytic BP measurements were recorded with the ABPM device at the aforementioned intervals. Recordings were included in the analysis if they had >80% of the total readings valid with a maximum of two non-consecutive (but not two consecutive) day-hours with fewer than two valid measurements and a maximum of one night-hour without valid recording. Patients with invalid recordings, repeated the 48-hour ABPM one week later while remaining on the same dialysate sodium. To minimize the possible effect of manual BP measurements, only measurements obtained at the time intervals at which the device was programmed to measure BP were used in this analysis.

Statistical analysis

Statistical analysis was performed with Statistical Package for Social Sciences version 22.0 (SPSS Inc, Armonk, NY, USA). Sample size calculation was performed based on the primary outcome, i.e., a difference in 48-hour SBP between low and standard dialysate sodium concentrations [17]. All continuous data are presented as mean ± standard deviation (mean±SD) as they followed the normal distribution, which was examined with the Shapiro-Wilk test. Categorical variables are expressed as frequencies and percentages (n, %). Paired Student t-test was used to examine differences between low versus standard dialysate sodium in peridialytic and ambulatory BP and BP changes from baseline. A p-value <0.05 (two-tailed) was considered statistically significant in all comparisons.

## Results

Baseline characteristics

**Figure 1:**
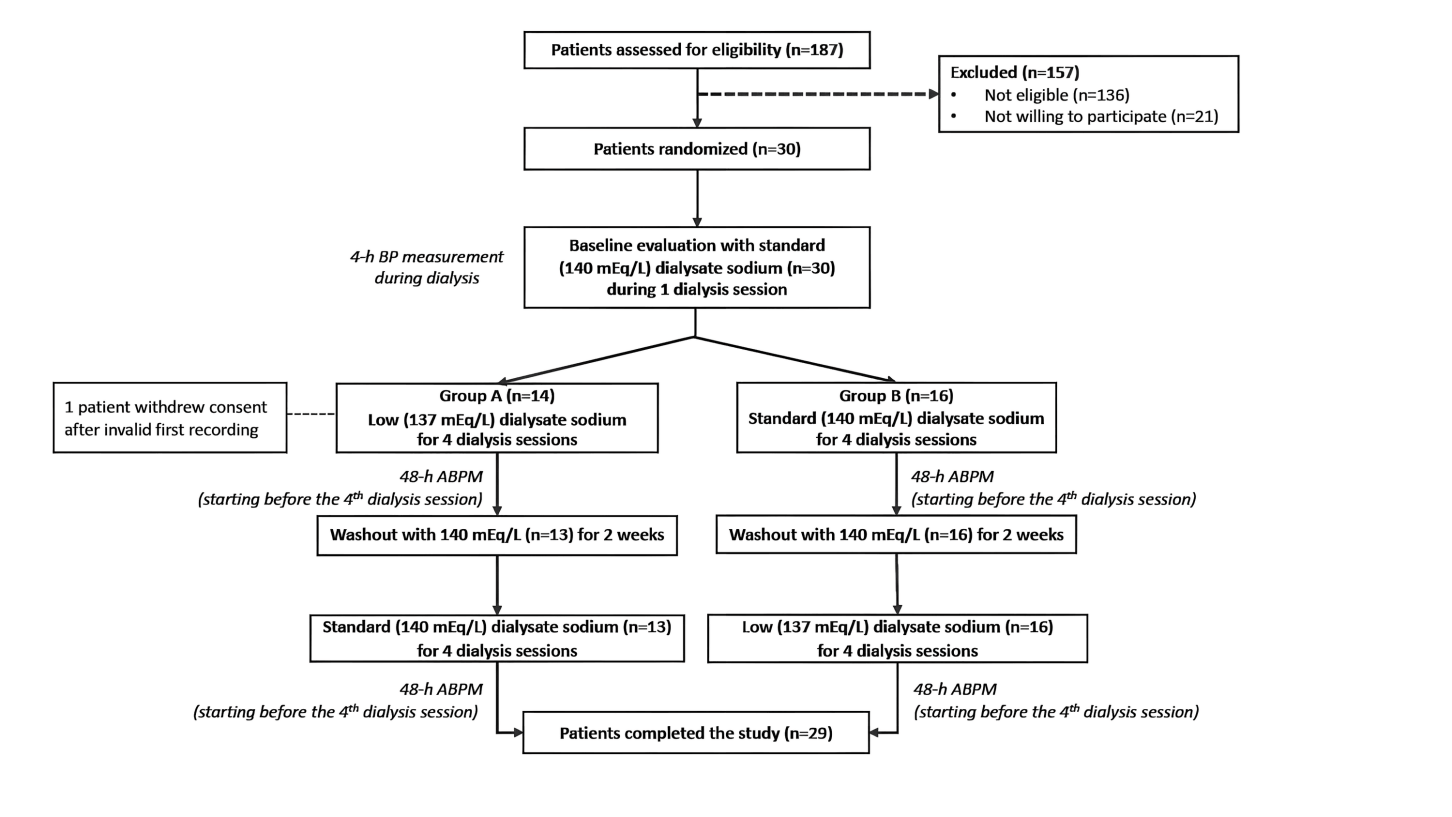
Study flowchart. Abbreviations: ABPM, ambulatory blood pressure monitoring; BP, blood pressure.

The flowchart of study participants is depicted in Figure 1. Baseline demographic, clinical, and laboratory parameters of the study population are presented in Table 1. A total of 29 participants (17 male and 12 female) were included in this analysis. They had a mean age of 64.6±17.6 years and a median hemodialysis vintage of 28.7 (8.3-69.8) months. Among major co-morbidities, all participants had hypertension, approximately 40% had diabetes mellitus, 37% had coronary heart disease and 40% had heart failure. With regards to antihypertensive medication, calcium channel blockers (CCBs) and β-blockers were the most common drug classes, both prescribed to 62% of the study population. Baseline pre-/post-dialysis and intradialytic aortic BP are also shown in Table 1. Ambulatory 48-hour SBP/DBP were significantly decreased with low compared to standard dialysate sodium concentration (137.6±17.0/81.4±13.7 mmHg with low vs. 142.9±14.5/84.0±13.9 mmHg with standard dialysate sodium, p=0.005/p=0.007 respectively). Of note, mean intradialytic weight gain (IDWG) was significantly lower with low (1.4±0.8 kg) than with standard dialysate sodium (1.8±0.7 kg, p=0.012).

**Table 1:** Baseline demographic, clinical and laboratory characteristics of the study population. Abbreviations: ACEIs, angiotensin converting-enzyme inhibitors; ARBs, angiotensin II receptor blockers; BMI, body mass index; CCBs, calcium channel blockers; DBP, diastolic blood pressure; ESKD, end-stage kidney disease; IDWG, intradialytic weight gain; SBP, systolic blood pressure

Parameter	Value
N	29
Male	17 (58.6%)
Age (years)	64.6±17.6
BMI (kg/m^2^)	24.58±4.78
Dialysis vintage (months)	28.70 (8.3, 69.8)
Dry weight (kg)	68.97±15.41
IDWG (kg)	1.7±0.9
Comorbidities	
Hypertension (n, %)	29 (100%)
Diabetes mellitus (n, %)	12 (41.4%)
Coronary Heart Disease (n, %)	11 (37.9%)
Heart failure (n, %)	12 (41.4%)
Smoking (n, %)	13 (44.8%)
Stroke (n, %)	3 (10.3%)
Antihypertensive medication	
ACEIs/ARBs (n, %)	11 (37.9%)
CCBs (n, %)	18 (62.1%)
β-blockers (n, %)	18 (62.1%)
Loop diuretics (n, %)	17 (58.6%)
Centrally active agents (n, %)	5 (17.2%)
α blockers (n, %)	11 (37.9%)
Pre-dialysis HR (beats/min)	70.9±10.3
Pre-dialysis aortic SBP (mmHg)	134.3±19.1
Pre-dialysis aortic DBP (mmHg)	89.7±14.5
Post-dialysis HR (beats/min)	68.8±9.7
Post-dialysis aortic SBP (mmHg)	149.5±20.1
Post-dialysis aortic DBP (mmHg)	101.4±18.6
Intradialytic HR (beats/min)	65.3±9.1
Intradialytic aortic SBP (mmHg)	132.6±17.0
Intradialytic aortic DBP (mmHg)	90.0±12.7

The effect of dialysate sodium concentration on aortic BP 

The levels of aortic SBP and DBP during the complete 48-hour, intradialytic, peridialytic and day-time/night-time periods with the two different dialysate sodium concentrations are presented in Table 2. Mean 48-hour aortic SBP was significantly lower with low compared to standard dialysate sodium concentration (124.1±16.4 vs 128.5±12.9 mmHg respectively, p=0.013). Similarly, patients had significantly lower 44-hour aortic SBP, pre-dialysis aortic SBP (126.4±17.4 vs 135.6±18.6 mmHg, p=0.044) and post-dialysis aortic SBP (137.0±20.0 vs 147.9±18.1 mmHg, p=0.01), and a trend for lower intradialytic SBP (127.7±17.5 vs 133.1±13.6 mmHg, p=0.158) with low compared to standard dialysate sodium. 

**Table 2:** The effect of dialysate sodium concentration on aortic blood pressure. Data are presented as mean±SD and comparisons are made with paired Student t-test for related samples. Abbreviations: DBP, diastolic blood pressure; SBP, systolic blood pressure.

Parameter	Low dialysate sodium	Standard dialysate sodium	t	p
48-h aortic SBP (mmHg)	124.1±16.4	128.5±12.9	-2.649	0.013
48-h aortic DBP (mmHg)	83.0±14.1	85.8±14.1	-2.950	0.006
Pre-dialysis aortic SBP (mmHg)	126.4±17.4	135.6±18.6	-2.126	0.044
Pre-dialysis aortic DBP (mmHg)	85.9±12.8	91.8±11.2	-3.084	0.005
Post-dialysis aortic SBP (mmHg)	137.0±20.0	147.9±18.1	-2.753	0.01
Post-dialysis aortic DBP (mmHg)	93.8±15.5	97.3±14.9	-1.636	0.114
Intradialytic aortic SBP (mmHg)	127.7±17.5	133.1±13.6	-1.973	0.158
Intradialytic aortic DBP (mmHg)	87.0±13.2	90.0±12.6	-2.325	0.028
44-h aortic SBP (mmHg)	123.7±16.7	127.8±13.9	-2.394	0.024
44-h aortic DBP (mmHg)	82.5±14.5	85.3±14.7	-2.662	0.013
Day-time aortic SBP (mmHg)	124.6±16.5	129.6±13.3	-3.035	0.005
Day-time aortic DBP (mmHg)	83.9±14.7	86.9±14.3	-2.981	0.006
Night-time aortic SBP (mmHg)	122.4±19.3	126.1±13.3	-1.360	0.185
Night-time aortic DBP (mmHg)	80.4±13.7	83.0±14.3	-1.775	0.087

With regards to DBP, 48-hour aortic DBP was significantly reduced with low compared to standard dialysate sodium concentration (83.0±14.1 vs 85.8±14.1 mmHg, p=0.006). Pre-dialysis and intradialytic aortic DBP levels were also significantly different between low and standard dialysate sodium (pre-dialysis: 85.9±12.8 vs 91.8±11.2 mmHg, p=0.005; intradialytic: 87.0±13.2 vs 90.0±12.6 mmHg, p=0.028), while post-dialysis aortic DBP was lower with low dialysate sodium but did not reach statistical significance (93.8±15.5 vs 97.3±14.9 mmHg, p=0.114). Τhe trajectories of aortic SBP/DBP with low and standard dialysate sodium during the complete 48-hour period are illustrated in Appendix 1.

Day-time aortic SBP/DBP were also significantly reduced with low compared to standard dialysate sodium concentration. With regards to the night-time period, a similar pattern of lower values with the low dialysate sodium was observed but relevant differences did not reach statistical significance.

The effect of dialysate sodium concentration on wave reflection and arterial stiffness indices

Table 3 shows 48-hour, intradialytic, peridialytic and day-time/night-time wave reflection and arterial stiffness parameters recorded. During the 48-hour period, AIx(75) was significantly lower with low compared to standard dialysate sodium (26.3±6.7 vs 27.7±5.8%, p=0.03), while there was a trend for lower PP and AP but not statistically significant. A similar pattern of significant differences in AIx(75) was also observed during the 44-hour and intradialytic period. Ambulatory 48-hour and 44-hour PWV was also significantly lower with low dialysate sodium (48-h: 9.9±2.5 vs 10.1±2.6 m/s, p=0.008).

**Table 3:** The effect of dialysate sodium concentration on wave reflection and arterial stiffness indices. Data are presented as mean±SD and comparisons are made with paired Student t-test for related samples. Abbreviations: AIx(75), heart rate-corrected augmentation index; AP, augmentation pressure; HR, heart rate; PP, pulse pressure; PWV, pulse wave velocity.

Parameter	Low dialysate sodium	Standard dialysate sodium	t	p
48-h interval				
HR (beats/min)	68.7±8.7	68.1±8.4	0.797	0.780
PP (mmHg)	41.1±8.5	42.7±7.0	-1.727	0.095
AIx(75) (%)	26.3±6.7	27.7±5.8	-2.284	0.03
AP (%)	13.5±5.2	14.7±4.3	-1.749	0.091
PWV (m/s)	9.9±2.5	10.1±2.6	-2.875	0.008
Pre-dialysis				
HR (beats/min)	68.6±11.5	69.2±9.5	0.116	0.826
PP (mmHg)	40.5±10.9	43.8±13.4	-1.065	0.297
AIx(75) (%)	27.3±12.9	32.1±11.2	-1.713	0.099
AP (%)	13.3±6.9	17.1±9.5	-2.099	0.046
PWV (m/s)	9.9±2.5	10.3±3.2	-2.594	0.016
Post-dialysis				
HR (beats/min)	71.7±14.1	70.6±12.7	0.409	0.762
PP (mmHg)	43.2±17.2	50.7±14.7	-2,176	0.038
AIx(75) (%)	25.4±16.2	30.6±13.3	-1.906	0.067
AP (%)	13.1±10.2	18.4±10.8	-2.517	0.018
PWV (m/s)	10.2±2.6	10.7±2.7	-2.863	0.008
Intradialytic				
HR (beats/min)	65.3±9.3	65.8±8.9	-0.540	0.829
PP (mmHg)	40.7±10.7	43.2±11.1	-1.287	0.209
AIx(75) (%)	23.9±8.8	26.5±7.4	-2.101	0.045
AP (%)	13.4±7.7	15.1±7.0	-1.340	0.191
PWV (m/s)	10.0±2.7	10.2±2.7	-1.879	0.071
44-h interval				
HR (beats/min)	69.1±8.7	68.4±8.5	0.922	0.746
PP (mmHg)	41.2±8.6	42.6±6.9	-1,493	0.147
AIx(75) (%)	26.5±6.8	27.8±5.9	-1.928	0.064
AP (%)	13.6±5.1	14.6±4.2	-1.524	0.139
PWV (m/s)	9.9±2.5	10.0±2.6	-2.638	0.013
Day-time				
HR (beats/min)	69.4±8.9	68.5±8.4	1.043	0.704
PP (mmHg)	40.7±8.0	42.7±7.3	-2.303	0.029
AIx(75) (%)	25.8±6.7	27.6±6.2	-2.729	0.011
AP (%)	13.0±4.9	14.7±4.9	-2.469	0.02
PWV (m/s)	9.9±2.5	10.1±2.6	-3.379	0.002
Night-time				
HR (beats/min)	66.9±9.3	66.8±10.0	0.084	0.974
PP (mmHg)	42.0±11.1	43.1±7.7	-0.692	0.495
AIx(75) (%)	27.4±8.4	28.3±6.1	-0.784	0.440
AP (%)	14.9±7.0	15.9±5.3	-0.952	0.349
PWV (m/s)	9.8±2.4	10.0±2.5	-1.355	0.186

With regards to pre- and post-dialysis wave reflection indices, AP was significantly reduced with low dialysate sodium (post-dialysis: 13.1±10.2 vs 18.4±10.8%, p=0.018), while differences in AIx(75) were consistent but not statistically significant. Of note, post-dialysis PP was considerably lower with the low dialysate sodium (43.2±17.2 vs 50.7±14.7 mmHg, p=0.038). Both pre- and post-dialysis PWV were significantly lower with low compared to standard dialysate sodium (post-dialysis: 10.2±2.6 vs 10.7±2.7 m/s, p=0.008).

During the day-time period, all arterial stiffness parameters examined were significantly lower with the low compared to standard dialysate sodium. In contrast, non-significant differences were observed in all of the above indices during the night-time period.

Changes from baseline in aortic BP and arterial stiffness parameters with low and standard dialysate sodium concentration

**Figure 2:**
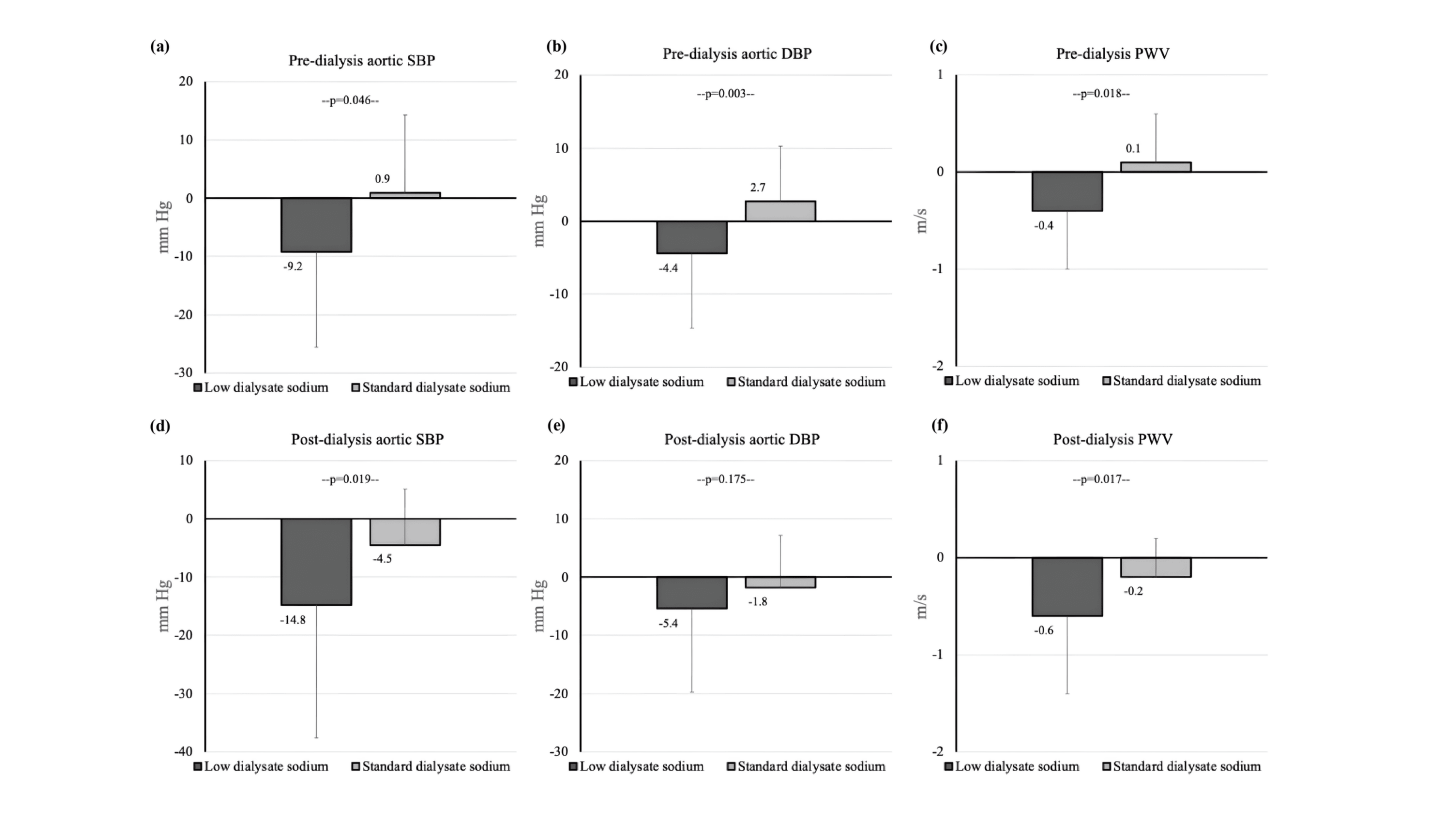
Changes from baseline in (a) pre-dialysis aortic SBP, (b) pre-dialysis aortic DBP, (c) pre-dialysis PWV, (d) post-dialysis aortic SBP, (e) post-dialysis aortic DBP and (f) post-dialysis PWV with low versus standard dialysate sodium concentration. Abbreviations: DBP, diastolic blood pressure; SBP, systolic blood pressure; PWV, pulse wave velocity.

The differences in the changes (Delta, Δ) in pre-, post- and mean intradialytic aortic BP and arterial stiffness parameters from baseline between low and standard dialysate sodium concentrations are presented in Appendix 2. Mean change in pre-/post-dialysis aortic SBP was significantly greater with low dialysate sodium compared to standard dialysate sodium concentration (pre-dialysis ΔSBP: -9.2±16.4 mmHg vs 0.9±13.4 mmHg, p=0.046; post-dialysis ΔSBP: -14.8±22.8 mmHg vs -4.5±9.6 mmHg respectively, p=0.019). Pre-dialysis aortic DBP displayed a significantly greater decrease with low dialysate sodium (ΔDBP: -4.4±10.3 mmHg with low vs 2.7±7.6 mmHg with standard dialysate sodium, p=0.003), while reduction in post-dialysis aortic DBP was not statistically different, though a trend for greater decrease was observed. Intradialytic aortic BP followed a similar pattern of numerical reduction with low vs standard dialysate sodium. Among the various arterial stiffness parameters examined, all indices showed a higher decrease from baseline with low dialysate sodium; however only the differences in changes in PWV reached statistical significance (change in pre-dialysis PWV: -0.4±0.6 vs 0.1±0.5 m/s, p=0.018; change in post-dialysis PWV: -0.6±0.8 vs -0.2±0.4 m/s, p=0.017, with low vs standard dialysate sodium, respectively). Figure 2 shows the changes from baseline in pre- and post-dialysis aortic SBP, DBP and PWV.

## Discussion

This is a prespecified secondary analysis of a randomized crossover study examining the effect of low versus standard dialysate sodium concentration on ambulatory aortic BP during a complete 48-hour period in patients with IDH. Similar to our findings regarding peripheral BP, mean 48-hour aortic SBP/DBP was significantly lower with low dialysate sodium compared with standard dialysate sodium concentration, with an observed mean difference of -4.4 (95%CI, -1.00 to -7.8) / -2.8 (95%CI, -0.9 to -4.7) mmHg. Pre- and post-dialysis aortic BP was also significantly reduced with low vs standard dialysate sodium. Overall, this pattern of differences between the two dialysate sodium concentrations was consistent across the 44-hour, day-time and night-time periods, with most comparisons showing statistical significance except for night-time BP. With regards to arterial stiffness, all indices examined followed the same direction of lower levels with low compared to standard dialysate sodium; among them, AIx(75) was significantly lower during the 48-hour, 44-hour, day-time and intradialytic period. Differences in peridialytic and ambulatory PWV were also evident. 

The mechanistic background of IDH is complex and involves interaction of several pathophysiological pathways, including increased arterial stiffness [11]. The physiological consequence of increased arterial stiffness is premature arrival of the reflected pulse wave from the periphery to the aorta (i.e., during systole rather than diastole), leading to a rise in aortic SBP [21]. In a previous study by Mourad et al. in 47 hemodialysis patients without cardiovascular disease, patients whose BP was unresponsive to ultrafiltration had significantly higher PWV compared to those with an intradialytic BP fall (12.9±2.7 vs. 10.8±2.9 m/s, p<0.05) and there was a positive correlation between PWV and intradialytic BP changes [22]. Similarly, in a subsequent cross-sectional study, patients with IDH exhibited higher carotid-femoral PWV and AIx compared with patients without this phenomenon [23]. In a study including 70 hemodialysis patients, we previously observed that those with IDH had significantly higher pre-dialysis PWV (10.4±1.6 vs 8.3±1.9 vs 9.4±2.4 m/s, p<0.01) and AIx(75) (28.1±7.3 vs 21.7±8.6 vs 25.8±8.2%, p<0.05), while the expected reduction in AIx(75) during dialysis was practically absent compared with individuals without IDH [14]. Finally, in another study of our group we showed that IDH patients exhibit significantly higher levels of ambulatory aortic BP and arterial stiffness parameters during a complete 48-hour period. These results support increased arterial stiffness as a key factor in the pathogenesis of IDH. Although the exact underlying mechanisms remain to be fully elucidated, one possibility is that increased arterial stiffness decreases baroreceptor sensitivity and contributes towards autonomic nervous system dysfunction [11]. Indeed, 75% of recurrent IDH episodes, rather than evoking a feedback baroreceptor-mediated bradycardia and hypotension, are associated with sympathetic overactivation and synchronous increases in heart rate, indicating feed-forward effects on BP due to baroreceptor dysfunction [24].

Studies on the effects of non-pharmacological BP-lowering strategies on aortic BP in dialysis patients are scarce. In an observational study by Guerin et al., dry weight adjustment and use of antihypertensive medication for BP control also reduced carotid-femoral PWV, leading to improved outcomes when BP and PWV reductions occurred in parallel [9]. Randomized trials in hypertensive hemodialysis patients that using dry-weight probing by bioimpedance spectroscopy [25] or lung-ultrasound [26] showed greater reductions in carotid-femoral PWV and ambulatory aortic BP and PWV compared to standard treatment. With regards to dialysate sodium, Liu et al. examined the effect of different sodium concentrations (136 vs 138 mEq/L) on carotid-femoral PWV and ambulatory BP [27]. PWV decreased in both groups at study end, with a trend for higher reduction with the lower dialysate sodium. However, the study had limitations, including confounding by antihypertensive medication changes and PWV measurement only in office conditions. 

In patients with IDH, studies examining the effects of BP-lowering strategies on aortic BP are extremely rare. In a non-randomized study by Inrig et al. [28], examining the effects of carvedilol in IDH, patients with resolution of IDH after treatment with carvedilol showed a non-significant decrease in PWV (12.4±3.5 vs. 11.8±3.0 m/s, p=0.3), while those with persistent IDH, had a non-significant increase in PWV after carvedilol administration (10.7±2.20 vs. 11.3±3.55 m/s, p=0.8). The only randomized study evaluating a pharmacological intervention on ambulatory aortic BP and arterial stiffness in IDH was a previous work from our group that showed one-week use of both nebivolol and irbesartan significantly reducing 24-hour aortic BP and 24-hour PWV [16]. 

The present study is the first to examine a non-pharmacological intervention, i.e. low dialysate sodium, on ambulatory aortic BP and arterial stiffness in patients with IDH. We demonstrated that low dialysate sodium, significantly reduced ambulatory aortic BP levels and PWV through the 48-hour interdialytic period. This is particularly important, since previous observations suggest that IDH patients have a profile of increased aortic BP and arterial stiffness during both the intra- and interdialytic period [15]. In addition, prior evidence indicates that arterial stiffness in the general population is primarily pressure-dependent and linked, apart from structural changes of the arterial wall, to functional factors like mechanical stress and angiotensin-II levels [29]. While BP reduction is typically accompanied by decreased arterial stiffness in the general population, patients with ESKD may have two different patterns of association between BP and arterial stiffness: one where BP changes largely modulate PWV levels (BP-dependent PWV), and another where BP changes are not associated with PWV (BP-insensitive PWV), i.e., arterial stiffness remains unchanged when BP is reduced [8]. The underlying mechanism for the later involves unique factors for arterial damage in this population (i.e. increased calcium-phosphate products and accelerated arterial calcification) [30]. Observational findings suggest that this BP-independent component of PWV greatly contributes to adverse cardiovascular outcomes [8,9]. Our study expands the above observations, showing lower ambulatory aortic BP and PWV levels in parallel to lower peripheral BP levels with low dialysate sodium concentration and identifying the presence of a BP-dependent part of PWV in this specific dialysis population.

This study has strengths and limitations. It followed a careful randomized cross-over design including a sufficient washout period of two weeks with standard dialysate sodium concentration, detailed patient monitoring, as well as a careful assessment of ambulatory BP and arterial stiffness parameters. A limitation of this analysis is that our sample size calculation was based on our primary outcome of 48-hour SBP; however, this has not affected our results considerably, as clear differences in all major parameters studied herein were evident. Additionally, we opted for a small difference between the two dialysate sodium concentrations compared, i.e. 3 mEq/L, in order to resemble more closely everyday practice; this may have masked higher between-group differences that could be noted with lower dialysate sodium concentrations. The duration of each dialysate sodium period may be relatively short; however, opting for longer intervention periods would demand longer washout periods and may increase the drop-out rates of enrolled patients. Finally, as we did not use applanation tonometry or invasive methods for determining aortic BP, wave reflection and arterial stiffness parameters and given that these parameters are estimated by the Mobil-O-Graph device using mathematical models which include brachial BP, further studies using direct methods of PWV measurement are welcome to confirm our observations.

## Conclusions

In conclusion, this study evaluated the effect of low compared to standard dialysate sodium concentration on 48-hour ambulatory aortic BP and arterial stiffness in patients with IDH. We showed that low dialysate sodium reduced ambulatory aortic BP and arterial stiffness parameters, such as AIx(75) and PWV, compared to standard dialysate sodium. These findings support the use of low dialysate sodium as a major therapeutic approach in IDH patients that may offer additional benefits of decreasing aortic BP and PWV, apart from ambulatory peripheral BP. Future long-term studies are needed to confirm the effect of low dialysate sodium on arterial stiffness reduction and examine its impact on major outcomes in these individuals. 

## Figures and Tables

**Figure 3:**
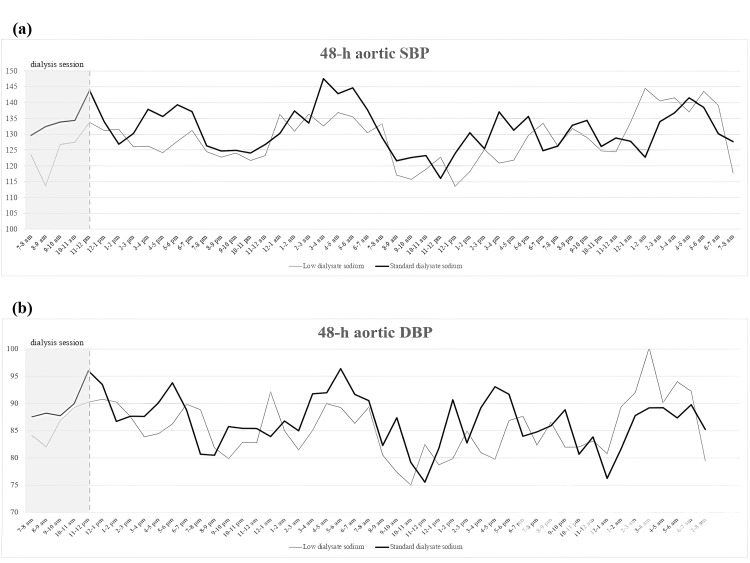
Trajectories of 48-h aortic BP with low and standard dialysate sodium concentration for (a) aortic SBP and (b) aortic DBP [data displayed for patients (N=8) in morning shift]. Abbreviations: DBP, diastolic blood pressure; SBP, systolic blood pressure.

## Appendices

Appendix 1

Appendix 2

**Table 4:** Changes from baseline in aortic blood pressure and arterial stiffness parameters with low and standard dialysate sodium concentration. Data are presented as mean±SD and comparisons are made with paired Student t-test for related samples. Abbreviations: AIx(75), heart rate-corrected augmentation index; AP, augmentation pressure; DBP, diastolic blood pressure; PP, pulse pressure; PWV, pulse wave velocity; SBP, systolic blood pressure.

Parameter	Lowdialysate sodium		Standard dialysatesodium		t		p
Pre-dialysis							
aortic SBP (mmHg)		-9.2±16.4		0.9±13.4		-2.122	0.046
aortic DBP (mmHg)		-4.4±10.3		2.7±7.6		-3.360	0.003
PP (mmHg)		-5.0±11.8		-1.9±11.1		-0.893	0.382
AIx(75) (%)		-1.8±11.5		2.5±15.8		-1.471	0.156
AP (%)		-2.5±7.1		1.0±9.9		-1.844	0.079
PWV (m/s)		-0.4±0.6		0.1±0.5		-2.571	0.018
Post-dialysis							
aortic SBP (mmHg)		-14.8±22.8		-4.5±9.6		-2.532	0.019
aortic DBP (mmHg)		-5.4±14.3		-1.8±9.0		-1.403	0.175
PP (mmHg)		-9.4±14.3		-2.7±10.6		-2.078	0.05
AIx(75) (%)		-4.7±20.8		-1.0±15.5		-1.256	0.222
AP (%)		-6.7±13.2		-2.7±9.9		-2.081	0.049
PWV (m/s)		-0.6±0.8		-0.2±0.4		-2.592	0.017
Mean intradialytic							
aortic SBP (mmHg)		-4.9±11.5		0.6±13.3		-1.973	0.058
aortic DBP (mmHg)		-2.8±9.4		0.2±7.3		-2.325	0.028
PP (mmHg)		-2.1±5.5		0.4±9.4		-1.287	0.209
AIx(75) (%)		-2.2±7.0		0.4±6.7		-2.101	0.045
AP (%)		-1.6±5.0		0.1±5.7		-1.340	0.191
PWV (m/s)		-0.2±0.5		0.0±0.5		-1.879	0.071
